# Selection of the optimal reference genes for expression analyses in different materials of *Eriobotrya japonica*

**DOI:** 10.1186/s13007-019-0391-2

**Published:** 2019-01-28

**Authors:** Wenbing Su, Yuan Yuan, Ling Zhang, Yuanyuan Jiang, Xiaoqing Gan, Yunlu Bai, Jiangrong Peng, Jincheng Wu, Yuexue Liu, Shunquan Lin

**Affiliations:** 1 Key Laboratory of Innovation and Utilization of Horticultural Crop Resources in South China (Ministry of Agriculture), College of Horticulture, South China Agricultural University, Guangzhou, 510642 China; 2 College of Horticulture, Shenyang Agricultural University, Shenyang, 110866 China; 3 Key Laboratory of Loquat Germplasm Innovation and Utilization, Putian University, Putian, 351100 China; 4 Guangzhou Institute of Agricultural Sciences, Guangzhou, 510308 China

**Keywords:** Loquat, RT-qPCR, Reference gene, Gene expression, Off-season fruit

## Abstract

**Background:**

Loquat (*Eriobotrya japonica*) is a subtropical tree bearing fruit that ripens during late spring and early summer, which is the off-season for fruit production. The specific flowering habit of loquat, which starts in fall and ends in winter, has attracted an increasing number of researchers who believe that it may represent an ideal model for studying flowering shift adaptations to climate change in Rosaceae. These studies require an understanding of gene expression patterns within the fruit and other tissues of this plant. Although *ACTIN*s (*ACT*s) have previously been used as reference genes (RGs) for gene expression studies in loquats, a comprehensive analysis of whether these RGs are optimal for normalizing RT-qPCR data has not been performed.

**Results:**

In this study, 11 candidate RGs (*RIBOSOMAL*-*LIKE PROTEIN4* (*RPL4*), *RIBOSOMAL*-*LIKE PROTEIN18* (*RPL18*), *Histone H3.3* (*HIS3*), *Alpha*-*tubulin*-*3* (*TUA3*), *S*-*Adenosyl Methionine Decarboxylase* (*SAMDC*), *TIP41*-*like Family Protein* (*TIP41*), (*UDP*)-*glucose Pyrophosphorylase* (*UGPase*), *18S ribosomal RNA* (*18S*), *Glyceraldehyde*-*3*-*phosphate Dehydrogenase* (*GAPDH*), *Plasma Intrinsic Protein 2* (*PIP2*) and *ACTIN*(*ACT*)) were assessed to determine their expression stability in 23 samples from different tissues or organs of loquat. Integrated expression stability evaluations using five computational statistical methods (GeNorm, NormFinder, ΔCt, BestKeeper, and RefFinder) suggested that a RG set, including *RPL4*, *RPL18*, *HIS3* and *TUA3,* was the most stable one across all of the tested loquat samples. The expression pattern of *EjCDKB1;2* in the tested loquat tissues normalized to the selected RG set demonstrated its reliability.

**Conclusions:**

This study reveals the reliable RGs for accurate normalization of gene expression in loquat. In addition, our findings demonstrate an efficient system for identifying the most effective RGs for different organs, which may be applied to related rosaceous crops.

**Electronic supplementary material:**

The online version of this article (10.1186/s13007-019-0391-2) contains supplementary material, which is available to authorized users.

## Introduction

Loquat (*Eriobotrya japonica* Lindl.) is a subtropical evergreen fruit tree belonging to the apple subfamily Maloideae of Rosaceae. Loquat is primarily cultivated in Southeast Asia and Mediterranean regions [1] and differs from temperate-zone relatives, such as apple, pear and peach, which normally flower in the spring, by flowering in fall or early winter [2]. Loquat fruits mature during the slack season of the fresh fruit market in late spring or early summer, and the nutritious and succulent fruits are attracting an increasing number of consumers worldwide [3]. In addition, loquat is also used in traditional Chinese medicine due to an abundance of therapeutic compounds and secondary metabolites in its leaves and other tissues, as well as the presence of anti-cancer compounds [4, 5]. Both its delicious fruit and ability to adapt to various subtropical climates have convinced breeders and researchers that loquat cultivation should be expanded [6]. However, certain disadvantages, such as the large seeds and tender flesh, may hinder loquat production due to a reduced edibility or shorter shelf life [7].

Many classic breeding approaches to improve this crop are underway [8, 9]. Moreover, considerable research has focused on the genetics and molecular mechanisms underlying specific biological traits/phenomena in loquat for exploiting molecular markers for assisted selection [10, 11] or identifying important genes that may be directly or indirectly used in future breeding endeavors [2, 12–15]. Overall, gene expression analysis is one of the most important approaches for identifying candidate genes related to specific biological characteristics. Multiple molecular techniques, including northern blotting, semiquantitative reverse transcription PCR, in situ hybridization and quantitative reverse transcription-PCR, are effective methods of investigating the expression characteristics of specific genes. Among these, RT-qPCR (Quantitative Real-Time Polymerase Chain Reaction) is the most widely used technique due to its advantages of precision, high sensitivity, flexibility and scalability [16, 17]. However, confounding results are usually obtained when a reference gene (RG) without stable expression is used in gene expression assays [18]; therefore, the stability of a RG is critical for generating reliable and accurate qPCR results [19, 20].

Although an optimal RG was once considered to be a gene stably expressed in various tissues under different experimental conditions [19], many researchers have suggested that the transcript levels of commonly used housekeeping genes can vary considerably under different conditions [21, 22]. Thus, an optimal RG might not always be suitable for all materials under different experimental conditions [21]. Accordingly, an increasing number of studies have been conducted to identify reliable RGs for various plant materials or different developmental stages [22], such as vascular development [23], somatic embryogenic culture [24], root development [25] and flower development [26]. In addition, multiple attempts have been made to select suitable housekeeping genes for materials under biotic and abiotic stresses in different plant species, such as potato [27], *Arabidopsis* [28], rice [29] and *Pyropia yezoensis* [30]. Considering that fruit is one of the most important commercial products of most crop plants, numerous stable RGs have been identified for quantitative expression analyses of fruit development in various crops, such as grape [31], banana [32], papaya [33], Chinese wolfberry [34], olive [35], orange [36], strawberry [37], plum [38], watermelon [39], peach [40] and tomato [41].

Considerable efforts have been made to clarify the molecular regulatory mechanisms of specific biological traits/phenomena related to loquat fruit, such as sorbitol metabolism [42], ethylene synthesis and signal transduction [43], flesh coloration [12], postharvest fruit development [15], flowering time regulation [2], volatile component formation in fruit [13] and fruit size formation [14]. Conversely, few RGs have been identified and applied to loquat. Of all known loquat housekeeping genes, *ACTIN*s (*ACT*s, including JN004223, JX089589, AB710173.1 and FJ481118) have been widely used as RGs in research on fruit pulp coloration [12], floral initiation [2] and fruit cell division [14]. Nonetheless, previous gene expression analyses of fruit development lasting approximately four months have occasionally revealed the fluctuating *ACT* expression levels. Such results suggest that *ACT*s are not always suitable for detecting expression related to long-duration fruit development or even other experimental conditions. However, reports focused on RG selection in loquat have not been reported. Thus, there is a need to identify the appropriate RGs for gene expression studies based on RT-qPCR assays of the development of fruit or other tissues/organs in loquat.

In this study, partial 3′-terminal sequences of 7 new candidate RGs were cloned and identified. RT-qPCR assays of 23 samples belonging to five sample sets (fruit, floral tissues, seed development, vegetative development and all samples) and specific primer pairs for 11 candidate RGs were performed, and expression stability was evaluated with five methods: geNorm, Normfinder, BestKeeper, ΔCt and RefFinder. Comprehensive reports of the five sample sets based on these five methods were generated. The expression levels of *EjCDKB1;2* (*Cyclin*-*dependent kinase B1;2*) in fruit during the cell division phase were normalized to the most stable RG set (*RPL4*, *RPL18*, *HIS3* and *TUA3*) and the least stable RG set (*SAMDC*, *PIP2* and *18S*), with results demonstrating the reliability of the newly selected optimal RGs. RGs identified in this study provide a useful and reliable resource for the accurate quantification of gene expression in loquat and furthermore illustrate an effective system for identifying the best RGs for different tissues/organs of this crop.

## Materials and methods

### Plant materials

*Eriobotrya japonica* cv. ‘Zaozhong-6' cultivated in the *Eriobotrya* germplasm resource preservation garden at the South China Agricultural University under normal management was used as the plant material. A total of 23 samples were collected for RG selection, including fruits belonging to five developmental stages, 7 floral tissues, ovules belonging to three developmental stages, seeds belonging to three developmental stages and 5 vegetative tissues (shown in Additional file 1: Table S1). Receptacles or fruits were collected every 7 days from 4 DBA (days before anthesis) to 77 DPA (days past anthesis) for cell division gene expression assays.

### Total RNA extraction and cDNA synthesis

The total RNA of the samples was extracted separately using an EASYspin Plus Plant RNA Extraction Kit (Aidlab, Beijing, China) according to the manufacturer’s instructions. cDNA synthesis was performed using a PrimeScript™ RT Reagent Kit (TaKaRa Bio, Shiga, Japan) according to the manufacturer’s instructions.

### Identification of candidate reference genes

Eleven RGs were selected to identify the most stably expressed housekeeping gene(s). Sequence information of four reported candidate RGs (*18S Ribosomal RNA* (*18S*), *Glyceraldehyde*-*3*-*phosphate* (*GAPDH*), *Plasma Intrinsic Protein 2* (*PIP2*) and *ACT*, under accession numbers AB636342.1, JQ731608.1, JX041626.1 and AB710173.1) were obtained from the National Center for Biotechnology Information (NCBI, Bethesda, MD, USA) database. Another seven candidate RGs, including *Ribosomal Protein L4* (*RPL4*), *Ribosomal Protein L18* (*RPL18*), *Histone H3.3* (*HIS3*), *Alpha*-*tubulin*-*3* (*TUA3*), *S*-*Adenosyl Methionine Decarboxylase* (*SAMDC*), *TIP41*-*like Family Protein* (*TIP41*) and (*UDP*)-*glucose Pyrophosphorylase* (*UGPase*), were selected based on previous reports in other plant species [24, 32, 34, 44–47]. Preliminary sequence information of the seven newly selected RGs was queried using our unpublished loquat genomic data. To verify and obtain detailed cDNA sequence information, specific primers were designed to amplify the sequence of the 3′ end. All primer pairs were supplied commercially (Sangon, Guangzhou, China). The target sequence fragments were isolated using a cDNA mixture of diverse ‘Zaozhong-6’ tissues as previously performed [14] and the primers were listed in Additional file 1: Table S2. The sequence information of these seven newly selected genes was submitted to GenBank under accession numbers MH196506-MH196512.

### RT-qPCR primer design and validation

Quantitative primers for the 11 genes were designed using the BatchPrimer3 program [48], and primer specificities were confirmed as previously described [18]. A standard curve using a series of gradient-diluted cDNAs was generated to calculate the gene-specific PCR amplification efficiency (E) and correlation coefficients (R^2^) for each gene, and four replications were performed. The amplification efficiencies of the candidate RGs were calculated using the following formula: E (%) = (10^−1/slope^ − 1) × 100. Detailed sequence information for all primer pairs is listed in Table 1. To verify the stabilities of the screened RGs, the expression pattern of *EjCDKB1;2* (Forward: 5′-CTCGGTTCGGCTCACTACTC-3′, Reverse: 5′-GCCAATCTCGCAAAGAAGAA-3′) in loquat fruit was detected using the most and least stable RGs as internal genes.

**Table 1:** Reference gene primer sequences and amplicon characteristics for each primer pair used in the RT-qPCR

Gene	Gene description	GenBank ID	Primer sequence (5′–3′)	Amplicon length (bp)	Amplicon Tm (°C)	Amplification efficiency (%)	Regression coefficient (R^2)^
*RPL4*	Ribosomal protein L4	MH196506	F: AGGTTCAGTCAGTCGTCAGGC	215	81.59	102.91	0.994
			R: GGCGGTAGCCTCCTCCTTAG				
*RPL18*	Ribosomal protein L18	MH196507	F: ATGGGATTTGGCTTCGTTATC	177	82.17	106.49	0.999
			R: AGAGTTTTGCTGGGATGGTG				
*HIS3*	Histone H3.3	MH196508	F: GTTTCCAGAGCCACGCGG	156	84.93	92.83	0.999
			R: CACGCTCACCCCTGATCCTC				
*TUA3*	Alpha-tubulin-3	MH196509	F: ATGGTATGATGCCCAGTGACACC	144	81.49	108.74	0.999
			R: GACGGTTATTGATGAAGTCAGGACTG				
*SAMDC*	s-Adenosyl methionine decarboxylase	MH196510	F: CAGCTGAGTTCTCCATAGCCTTG	162	83.99	99.05	0.999
			R: AATCATCCTTGACAAAGCGGTG				
*TIP41*	TIP41-like family protein	MH196511	F: TGATGGGGCACTAATGAGGC	202	81.07	126.43	0.996
			R: GTCTTATGCATGATCACAGGAAGC				
*UGPase*	(UDP)-glucose pyrophosphorylase	MH196512	F: ACATTACAAGATGGCTTTGTTACCC	157	79.94	93.33	0.996
			R: CAGAAACCTTAAGGCTATCAAGCTC				
*18S*	18S ribosomal RNA	AB636342.1	F: AAGTCGTAACAAGGTTTCCGTAG	80	80.10	118.84	0.999
			R: CCGATTCTCTGGTCGTTCTG				
*GAPDH*	Glyceraldehyde-3-phosphate dehydrogenase	JQ731608.1	F: TACAGTTCCCGTGTGGTTGA	139	79.90	104.97	0.998
			R: CGAGAGGACGCAAGATAACA				
*PIP2*	Plasma intrinsic protein 2	JX041626.1	F: ATCATCGGCACCTTCGTC	124	85.29	94.92	0.997
			R: GCACAATAAACACAGCAAACC				
*ACT*	Actin	AB710173.1	F: CTTTCCCTCTATGCCAGTG	122	80.58	105.39	0.999
			R: CAAGGTCAAGCCTCAAGAT				

### Quantitative real-time polymerase chain reaction analysis

All RT-qPCRs were performed as described previously [14] using a LightCycler480 system with LC480 software (Roche Diagnostics, Penzberg, Germany). Each reaction contained 1 μL of diluted cDNA, 5 μL of iTaq™ universal SYBR Green Supermix (Bio-Rad, Foster City, CA, USA), 0.5 μL of each primer and 3 μL of ultrapure water to a final volume of 10 μL. The reactions were performed in 384-well reaction plates using the following conditions: 95 °C for 5 min, followed by 40 cycles of 95 °C for 15 s, 60 °C for 30 s, and 72 °C for 30 s. The melting curves were analyzed at 60–95 °C after amplification. Four independent replicates were performed when assessing the expression stability of the RGs, whereas three biological replicates were performed when examining the expression patterns of fruit quality-related genes.

### Data analysis

The Ct values of each RG were used to evaluate their expression levels. Expression stability was analyzed using the geNorm [49], NormFinder [50], BestKeeper [51] and ΔCt methods [52]. A comprehensive ranking report of the reliability of the genes was ultimately obtained using the RefFinder tool (http://150.216.56.64/ referencegene.php). The comprehensive stability value according to RefFinder was based on the NormFinder, BestKeeper, GeNorm, and comparative ΔCt results. The overall final ranking of each gene was computed as the geometric mean, and a lower geometric mean of the ranking value indicated a higher stability. SigmaPlot 12.5 software was used to prepare figures indicating the gene expression patterns.

## Results

### Isolation of loquat candidate reference genes

A total of 11 genes were selected for suitable RG identification for use in the expression analyses of different loquat materials. Four RGs, *18S*, *GAPDH*, *PIP2* and *ACTIN,* which have been reported in previous studies, were included. Considering the deficiency of appropriate RG information for loquat, *RPL4*, *RPL18*, *HIS3*, *TUA3*, *SAMDC*, *TIP41* and *UGPase*, which are often used as RGs for other plants, were also chosen for assessment as candidate loquat RGs in this study. Partial mRNA sequences (279–381 bp) from the 3′ end of these newly selected candidate genes were cloned and sequenced using primer pairs listed in Additional file 1: Table S2. Detailed sequence information can be found in GenBank under accession numbers MH196506 to MH196512.

### Verification of amplification efficiency

**Fig. 1:**
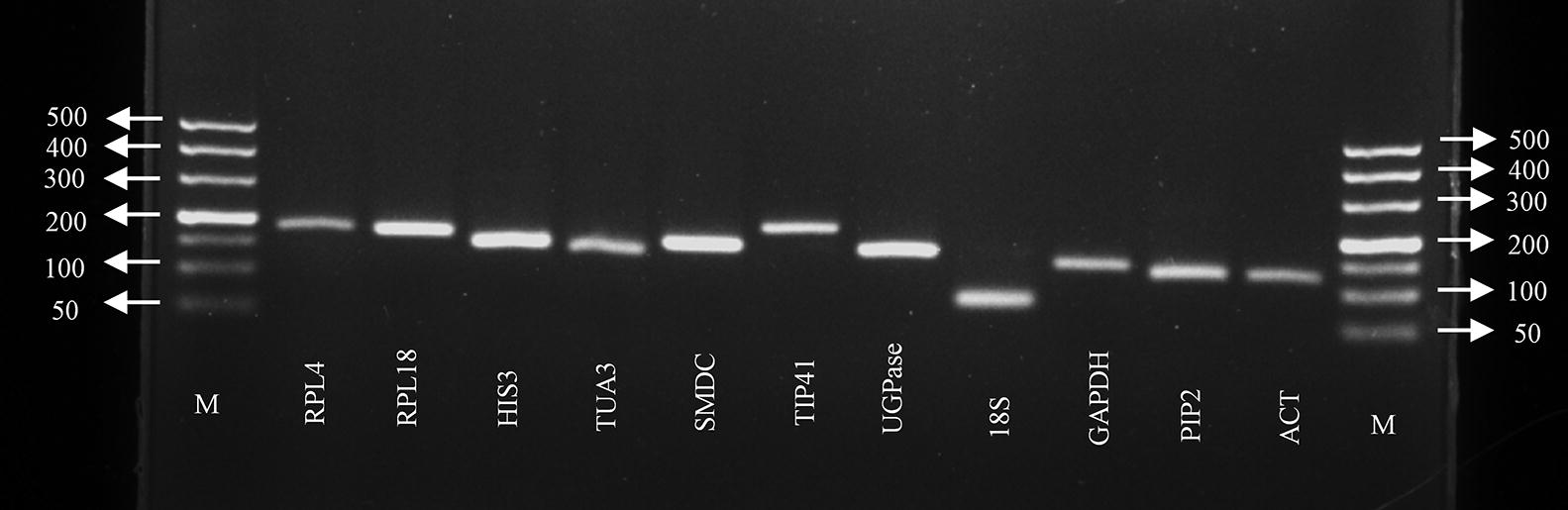
Specificity of the PCR and amplicon product length of each quantitative primer pair. Specific products of the expected size for each reference gene fragment after 2.0% agarose gel electrophoresis. M represents the DNA size marker

To ensure the specificity of our RT-qPCR analysis, primers for quantitative PCR of the 11 candidate RGs were designed to amplify the 3′ end of each gene, and the products were examined via agarose gel electrophoresis. The results showed that only the expected product was amplified for each of the tested RGs. The product sizes ranged from 80 bp (*18S*) to 215 bp (*RPL4*) (Fig. 1, Table 1). In addition, a single peak in the melting curve further supported specific amplification by each primer pair (Additional file 1: Figure S1). The amplification efficiencies of the genes ranged from 93.33% (*UGPase*) to 126.43% (*TIP41*). The primer sequences and amplification characteristics of all genes tested are summarized in Table 1.

### Expression stability of candidate RGs based on expression profiles

**Fig. 2:**
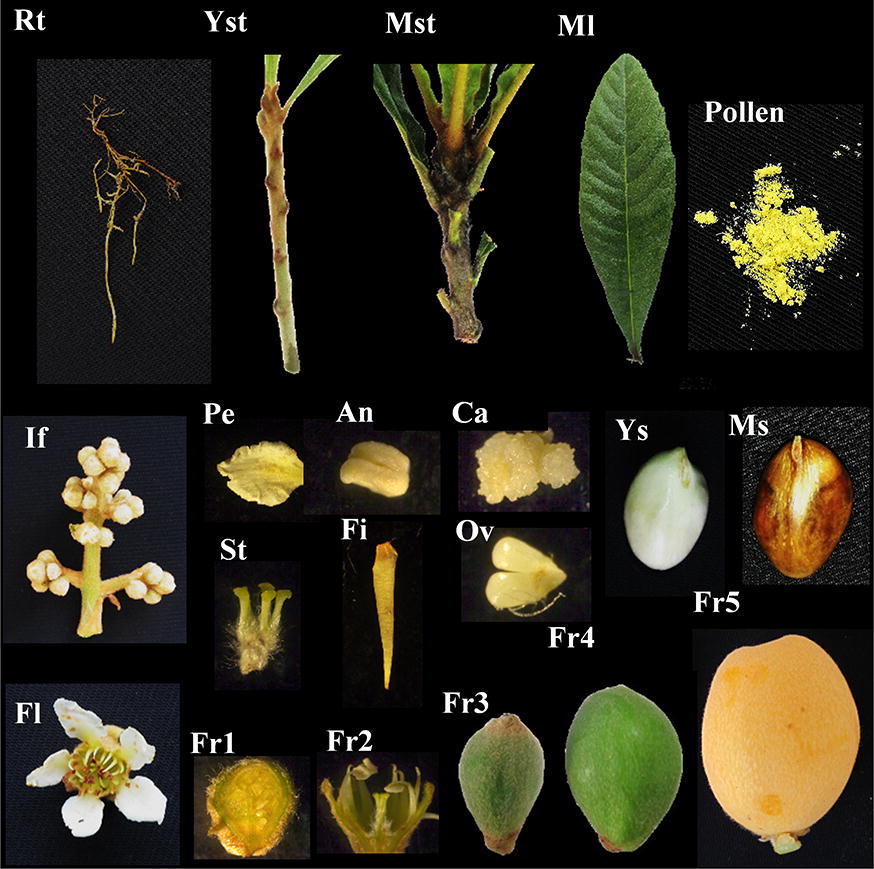
Loquat samples used in this study. Rt, root; Yst, young stem; Mst, mature stem; Ml, mature leave; If, inflorescence; Pe, petal; An, anther; St, stigma; Fi, filament; Ca, callus; Ov, ovule; Ys, young seed; Ms, mature seed; Fl, flower; Fi, filament; Fr1, fruit1, receptacle 21 days before anthesis; Fr2, fruit2, receptacle at anthesis; Fr3, fruit3, 56 days past anthesis; Fr4, fruit4, 102 days past anthesis; and Fr5, fruit5, mature fruit 126 days past anthesis

A total of 23 samples, including fruits, floral tissues, ovules, seeds and vegetative tissues at diverse developmental stages, were used for quantitative detection (Fig. 2, Additional file 1: Table S1). Our former expression assays of genes related to flowering time and fruit growth in loquat suggested that the use of one RG (*ACT2* or *ACT4*) for gene expression detection in multiple materials may not be suitable, none of *ACT*s exhibited stable expression in different tissues or the same tissues under various experimental conditions [2, 14]. Thus, to evaluate whether the candidate RGs are suitable for expression analysis in various developmental processes of loquat, the samples were divided into four subsets: fruit growth and quality development (Fruits), floral tissue development (Floral tissues), seed development (Ovules and seeds) and vegetative growth (Vegetative tissues).

**Fig. 3:**
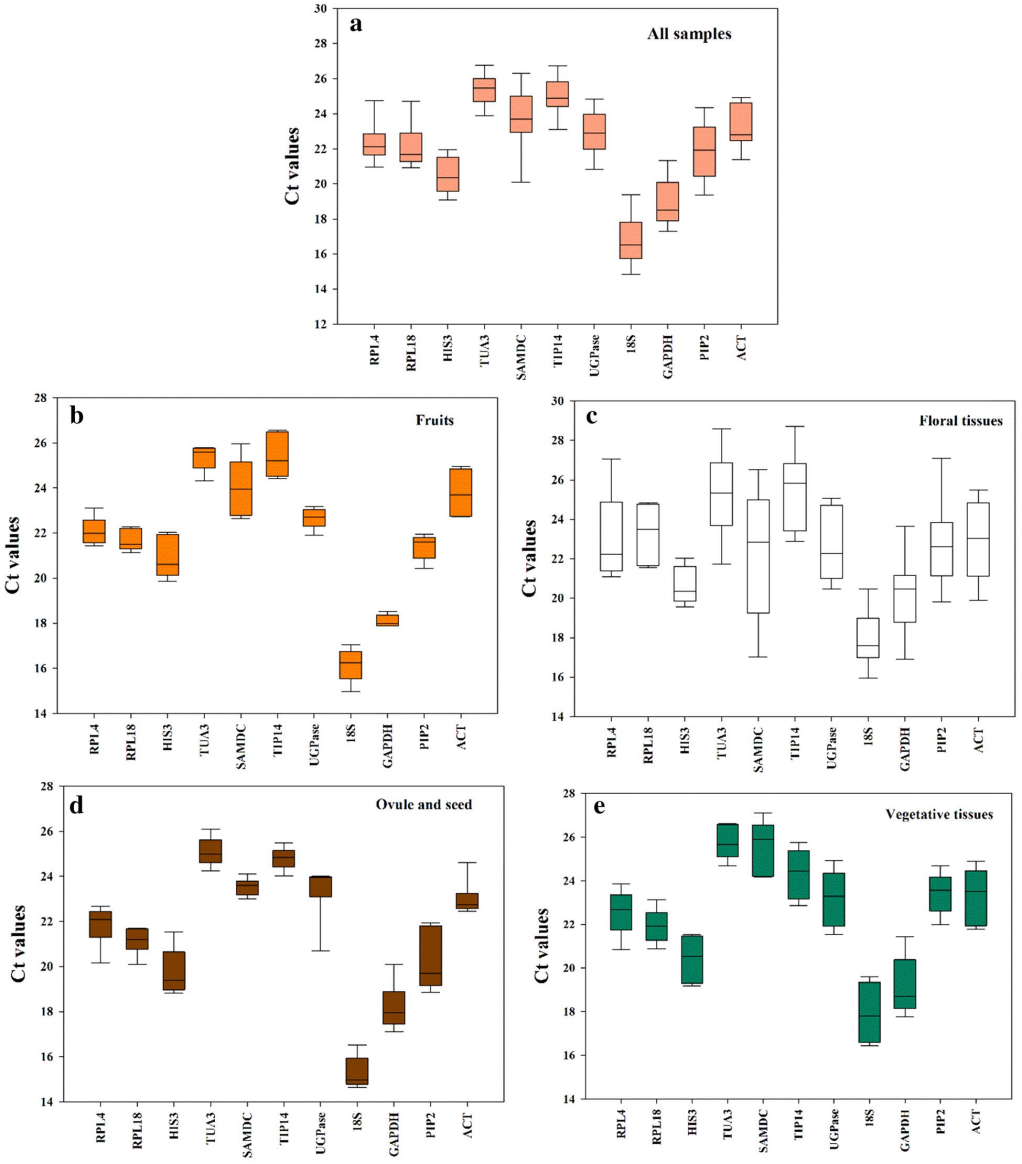
Boxplot analysis of the expression profiles of 11 candidate reference genes in 23 samples. The line across the box represents the median. The boxes represent the 25/75 percentiles. The whiskers show the maximum and minimum values

The expression levels of the 11 candidate RGs were first evaluated according to Ct values across all samples in an integrated set. As shown in the boxplot (Fig. 3a), the Ct values of these genes varied from 16.89 (*18S*) to 25.34 (*TUA3*). *TUA3* displayed the lowest expression, whereas *PIP2* showed the highest. *ACT*, the RG commonly used in former studies [2, 12, 14], showed a moderate expression level.

With respect to loquat fruit development, the Ct values of the different RGs ranged from 17.03 (*18S*) to 26.57 (*TIP41*). *SAMDC* and *GAPDH* showed the highest and lowest expression, respectively (Fig. 3b). According to the ΔCt values, *RPL18* had a minimum average STDEV, followed by *GAPDH* and *RPL4*; in contrast, *SAMDC* had the highest STDEV at 1.48 (Table 2). The NormFinder analysis found that *RPL18* was the most stable RG and *SAMDC* was the least stable (Table 2). Conversely, *TIP41*/*ACT* and *GAPDH* were the optimal RGs according to both the geNorm and BestKeeper analyses (Table 2). Finally, a comprehensive analysis considering all the methods using RefFinder suggested that *RPL18*, *GAPDH* and *TIP41* were the most stable RGs in loquat fruits (Table 2).

**Table 2:** Expression stability rankings of candidate reference genes evaluated by ΔCt, BestKeeper, NormFinder, geNorm, and RefFinder

Method	Expression stability Ranking order (1st is the most stable and 11th is the least stable)										
	*RPL4*	*RPL18*	*HIS3*	*TUA3*	*SAMDC*	*TIP41*	*UGPase*	*18S*	*GAPDH*	*PIP2*	*ACT*
*All samples*											
Delta CT	1.36 (1)	1.36 (2)	1.37 (3)	1.38 (4)	2.20 (11)	1.46 (6)	1.53 (8)	1.63 (9)	1.49 (7)	1.83 (10)	1.40 (5)
BestKeeper	1.03 (5)	0.98 (3)	0.87 (1)	0.91 (2)	1.50 (11)	1.01 (4)	1.06 (6)	1.29 (9)	1.25 (8)	1.43 (10)	1.10 (7)
NormFinder	0.79 (2)	0.84 (4)	0.80 (3)	0.78 (1)	1.99 (11)	1.02 (7)	1.05 (8)	1.25 (9)	1.02 (6)	1.51 (10)	0.87 (5)
geNorm	0.80 (1)	0.80 (1)	1.00 (4)	1.13 (6)	1.55 (11)	0.89 (3)	1.19 (7)	1.31 (9)	1.24 (8)	1.40 (10)	1.09 (5)
Recommended comprehensive ranking	1.78 (1)	2.21 (2)	2.45 (3)	2.63 (4)	11.00 (11)	4.74 (5)	7.20 (7)	9.00 (9)	7.20 (8)	10.00 (10)	5.44 (6)
*Fruit*											
Delta CT	0.83 (5)	0.67 (1)	0.85 (6)	0.86 (7)	1.48 (11)	0.83 (4)	1.02 (9)	0.82 (3)	0.72 (2)	1.10 (10)	0.89 (8)
BestKeeper	0.43 (6)	0.40 (3)	0.79 (8)	0.43 (5)	0.94 (11)	0.83 (9)	0.31 (2)	0.50 (7)	0.22 (1)	0.41 (4)	0.86 (10)
NormFinder	0.54 (4)	0.18 (1)	0.59 (7)	0.57 (5)	1.37 (11)	0.59 (6)	0.78 (9)	0.53 (3)	0.27 (2)	0.94 (10)	0.66 (8)
geNorm	0.61 (7)	0.43 (4)	0.31 (3)	0.64 (8)	0.92 (11)	0.20 (1)	0.73 (9)	0.53 (5)	0.59 (6)	0.79 (10)	0.20 (1)
Recommended comprehensive ranking	5.38 (6)	1.86 (1)	5.63 (7)	6.12 (8)	11.00 (11)	3.83 (3)	6.18 (9)	4.21 (4)	2.21 (2)	7.95 (10)	5.03 (5)
*Floral tissues*											
Delta CT	1.65 (5)	1.40 (2)	1.44 (3)	1.71 (8)	2.41 (11)	1.70 (7)	1.31 (1)	1.79 (9)	1.67 (6)	1.95 (10)	1.55 (4)
BestKeeper	1.87 (10)	1.18 (2)	0.82 (1)	1.69 (9)	2.57 (11)	1.58 (6)	1.33 (1)	1.20 (3)	1.64 (8)	1.59 (7)	1.56 (5)
NormFinder	1.16 (5)	0.79 (2)	0.81 (3)	1.22 (7)	2.21 (11)	1.32 (8)	0.36 (1)	1.41 (9)	1.19 (6)	1.58 (10)	0.95 (4)
geNorm	1.02 (5)	0.59 (1)	0.59 (1)	1.45 (9)	1.69 (11)	0.93 (4)	0.77 (3)	1.27 (7)	1.16 (6)	1.53 (10)	1.36 (8)
Recommended comprehensive ranking	5.95 (5)	1.68 (1)	1.73 (2)	8.21 (9)	11.00 (11)	6.05 (6)	1.86 (3)	6.42 (7)	6.45 (8)	9.15 (10)	5.03 (4)
*Ovule and seed*											
Delta CT	0.95 (8)	0.95 (7)	1.07 (9)	0.76 (2)	0.91 (5)	0.74 (1)	1.31 (10)	0.90 (4)	0.92 (6)	1.44 (11)	0.78 (3)
BestKeeper	0.62 (7)	0.40 (3)	0.79 (9)	0.49 (4)	0.30 (1)	0.34 (2)	0.91 (10)	0.56 (6)	0.73 (8)	1.09 (11)	0.54 (5)
NormFinder	0.73 (8)	0.70 (7)	0.87 (9)	0.16 (2)	0.60 (6)	0.12 (1)	1.22 (10)	0.59 (5)	0.57 (4)	1.37 (11)	0.27 (3)
geNorm	0.56 (5)	0.49 (3)	0.79 (9)	0.54 (4)	0.38 (1)	0.38 (1)	0.87 (10)	0.73 (8)	0.68 (7)	0.98 (11)	0.62 (6)
Recommended comprehensive ranking	6.88 (8)	4.58 (5)	9.00 (9)	2.83 (3)	2.34 (2)	1.19 (1)	10.00 (10)	5.57 (6)	6.05 (7)	11.00 (11)	4.05 (4)
*Vegetative tissues*											
Delta CT	0.83 (2)	0.78 (1)	1.08 (7)	0.92 (4)	1.43 (11)	0.91 (3)	1.01 (5)	1.22 (9)	1.23 (10)	1.18 (8)	1.03 (6)
BestKeeper	0.69 (4)	0.51 (1)	0.89 (5)	0.61 (2)	1.03 (9)	0.91 (6)	1.00 (8)	1.12 (11)	0.99 (7)	0.65 (3)	1.06 (10)
NormFinder	0.32 (2)	0.20 (1)	0.83 (7)	0.51 (3)	1.26 (11)	0.60 (4)	0.67 (5)	0.97 (9)	1.01 (10)	0.95 (8)	0.80 (6)
geNorm	0.47 (3)	0.54 (4)	0.65 (6)	0.62 (5)	1.06 (11)	0.21 (1)	0.74 (7)	0.97 (10)	0.91 (9)	0.84 (8)	0.21 (1)
Recommended comprehensive ranking	2.63 (2)	1.41 (1)	6.19 (7)	3.31 (4)	10.46 (11)	2.91 (3)	6.12 (6)	9.72 (10)	8.91 (9)	6.26 (8)	4.36 (5)

Values in Delta CT are the average STDEV and CV ± SD values for BestKeeper (CV, the coefficient of variance; SD standard deviation). Values in geNorm are expression stability values (M value), and values in NormFinder are stability values (SVs). Ranking values in RefFinder are geometric means of ranking values of the four computational programs. Values in brackets show the ranking orders of each gene

Of the 7 floral tissues examined, *TIP41* had the greatest Ct value at 25.55, whereas *GAPDH* had the lowest Ct value at 20.16 (Fig. 3c). Compared to other material sets, all candidate genes exhibited relatively higher Ct values in the floral tissues. The results of all evaluation methods indicated that *SAMDC* was the least stably expressed RG. *UGPase* was the most stable in the ΔCt and NormFinder assays, whereas *HIS3* or *HIS3*/*RPL18* was the most reliable housekeeping gene in the BestKeeper and geNorm evaluations. A comprehensive analysis of all evaluation methods using RefFinder revealed that *RPL18*, *HIS3* and *UGPase* were the top three most reliable RGs for expression studies of floral tissues (Table 2).

Regarding seed development, *TIP41* was found to be the most stable RG, and it had M values of 0.74, 0.12 and 0.38 in the ΔCt, NormFinder and geNorm assessments, respectively, whereas *SAMDC* was the best candidate according to BestKeeper (Tables 1, 2). In this sample set, *TUA3* showed the highest Ct value and *18S* showed the lowest value. The largest and smallest Ct value ranges were observed for *PIP2* and *SAMDC*, respectively (Fig. 3d). Overall, the comprehensive ranking order indicated that *TIP41*, *SAMDC and ACT* were the best RGs for studies on seed development.

When assessing materials related to vegetative growth, *RPL18* was the most stable RG, and it presented M values of 0.78, 0.51 and 0.20 according to the ΔCt, BestKeeper and NormFinder analysis, respectively, whereas *TIP41*/*ACT* was the most reliable RG according to the geNorm analysis. In most cases, *RPL4* was the second most reliable RG (Table 2), and *SAMDC* and *18S* were the least stable RGs according to all of the evaluation systems. An integration of the results of all four software analyses in RefFinder indicated that *RPL18, RPL4, TIP41* and *TUA3* were the most stably expressed RGs for studies on vegetative development in loquat.

Across all 23 samples, *RPL4* and *RPL18* were the most stable RGs using ΔCt and geNorm, whereas *HIS3* and *TUA3* were suggested to be the most reliable RGs using BestKeeper and NormFinder (Table 2). The comprehensive ranking by RefFinder further showed that *RPL4*, *RPL18*, *HIS3* and *TUA3* were the top four most stably expressed RGs, while *SAMDC*, *PIP2* and *18S* were the least stable RGs across all tested loquat samples. Former researches suggested that the use of a RG set, instead of one single housekeeping gene, would guarantee more accurate gene expression analyses under multiple experimental conditions [22], a RG set including the above selected stable RGs was recommended for the gene normalization in different materials of loquat.

### Validation of candidate reference genes

Although *ACT* has been commonly used as an RG in previous gene expression analyses in loquat [2, 12, 14], our results suggested that the RG set *RPL4*, *RPL18*, *HIS3* and *TUA3* is more suitable for gene expression analyses in loquat (Table 2). To confirm the reliability of this RG set, the expression pattern of *EjCDKB1;2*, which is described as a cell division marker gene [14], was assessed in diverse tissue samples.

When using the most stable RG set (*RPL4*, *RPL18*, *HIS3* and *TUA3*) for gene expression normalization, a single expression peak of *EjCDKB1;2* was observed during the fruit cell division phase. The transcription abundance of *EjCDKB1;2* dramatically increased soon after flowering, peaked at 28 DPA and then declined sharply until 42 DPA, when cell division of the pericarp was almost complete [14].

**Fig. 4:**
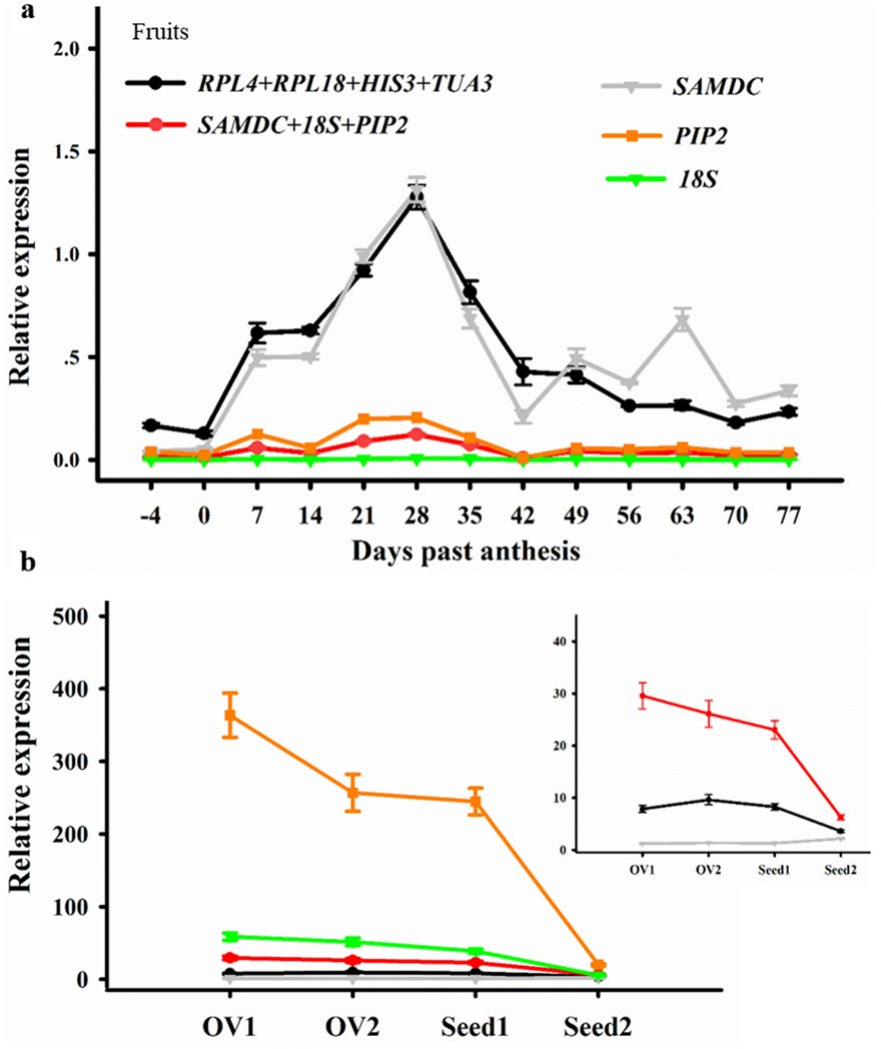
Relative expression levels of *EjCDKB1;2* normalized to the most and least stable RG sets. **a** Fruit development and **b** seed development. OV1 and OV2 were ovules in flowers of 4 and 1 day before anthesis. The most stable RG set included *RPL4*, *RPL18*, *HIS3* and *TUA3*. The least stable RGs included *SAMDC*, *PIP2* and *18S*

In contrast, distorted *EjCDKB1;2* expression results were obtained when using the least stable RGs as internal standards. When normalized to *PIP2*, *18S* or *SAMDC *+ *PIP2 *+ *18S*, an unanticipated decrease in *EjCDKB1;2* expression occurred from 7 to 14 DPA, and when normalized to *SAMDC*, a peak of *EjCDKB1;2* expression occurred at the end of the cell division phase (Fig. 4a).

Because of the limited number of reports focused on cell division in loquat tissue samples, only the seeds were used as another sample set for further verification. When the *RPL4 *+ *RPL18 *+ *HIS3 *+ *TUA3* combination was used as the RG, moderate *EjCDKB1;2* expression was obtained with small error bars. In addition, based on this set of the most stable RGs, an increase of *EjCDKB1;2* transcription abundance from OV1 to OV2 during prefertilization ovule development could be detected (Fig. 4b). Taken together, the results of *EjCDKB1;2* expression during fruit and seed development confirmed that *RPL4*, *RPL18*, *HIS3* and *TUA3* constitute the most appropriate set of RGs for accurate RT-qPCR analyses in loquat.

## Discussion

Loquat sets fruit in fall or early winter and as such provides appealing, nutritious and succulent pome fruits in the off-season during spring to early summer [1]; thus, it is a promising fruit tree for cultivation worldwide in the next decades [3]. In addition to classical breeding, molecular studies aim to develop or identify molecular markers/genes associated with specific traits that contributes to improving this fruit. *ACT*s have been commonly used as housekeeping genes [12–15]; however, stable RGs in loquat have not been comprehensively identified. In addition, an increasing amount of evidence has revealed that typical house-keeping genes exhibit variations among long-duration developmental samples of fruit and shoot apical meristem tissues [2, 14], which indicates that unclear results may be obtained when only one internal gene is applied under all experimental conditions. Thus, to avoid such an issue, selecting suitable RGs for these development phases and other tissues is critical.

In the present study, seven novel candidate house-keeping genes that have been commonly used in previous research on other plant species were selected as candidate RGs in loquat [24, 32, 34, 44–47]. Another four previously assessed housekeeping genes, *18S*, *GAPDH*, *PIP2* and *ACTIN*, were also evaluated. The expression stability of these genes in five sample subsets, which included 23 samples, was analyzed. Although NormFinder and geNorm are widely used to identify suitable RGs [32, 33, 53, 54], the computational method of RefFinder [55] was also employed in this study as formerly performed [26, 56, 57].

Reports have suggested that researchers should exercise caution when using ribosomal genes as RGs because these genes can display tissue specific expression (e.g. RPL39-like and RPL3-like [58]). However, many other ribosomal genes are also confirmed to be suitable housekeeping genes for their stable expression in all types of cells [59]. The stability of *RPLs* should be confirmed individually. The results in this study revealed that the loquat *RPL4/18* genes were stably expressed in all tissues examined (Table 2), which is consistent with the results of previous studies; for example, the stable expression of *RPL18* has been reported in grape and olive [60, 61]. In addition, many other plant ribosomal protein genes have been identified as suitable housekeeping genes for leaf and fruit samples, such as *MdRPL2* in apple [62], *CmRPL* in melon [54] and *MaRPS2* in banana [32]. Consistent with the stably expressed *HISTONE* in strawberry fruit [37] and *Taihangia* flower [26] and *CitTUA3* in citrus somatic embryo [24], the *HIS3* and *TUA3* genes in loquat were the 3rd and 4th most stable RGs across all the tested loquat samples (Table 2). *TIP41* encodes a tonoplast intrinsic protein, and its homologs in cucumber tissues [63] and melon fruit [54] display remarkably stable expression patterns, and the results of the present study indicated that it was the most suitable internal gene for seed development in loquat (Table 2).

To validate the accuracy of the stably expressed RGs identified in this study, the expression profile of *EjCDKB1;2*, which is regarded as a marker gene representing the cell division capacity of loquat [14], was detected during early fruit and seed development. The application of two or more stable reference genes might be more reliable than one single housekeeping gene for gene quantification [22]. In this study, the expression profile of *EjCDKB1;2* in fruit samples at different development stages was more consistent with the fruit cell division dynamics [14] when normalized to our newly identified RG set than when normalized to the least stable RGs (*SAMDC*, *PIP2* and *18S*) (Fig. 4a). Moreover, the most suitable RG set was able to detect a slight increase in *EjCDKB1;2* expression from OV1 to OV2 (Fig. 4b). Taken together, these results further confirmed that the most stable RGs, including *RPL4*, *RPL18*, *HIS3* and *TUA3*, identified by the comprehensive analyses are appropriate reference genes for accurate expression normalization in loquat.

## Conclusions

In this study, we identified a stably expressed RG set (including *RPL4*, *RPL18*, *HIS3* and *TUA3*) from 11 candidate internal genes with 23 loquat (*E. japonica*) tissue samples. An expression analysis of *EjCDKB1;2* in fruits and seeds further confirmed the suitability of the novel RG set. The findings provide a foundation for more accurate gene expression studies based on RT-qPCR in loquat. The findings can also be potentially transferred to closely related rosaceous crops and other agronomically important crops.

## Additional file

**Additional file 1: Table S1.** Samples used for reference gene selection. **Table S2.** Primer sequences for reference gene isolation. **Figure S1.** Melt curve analysis of the selected 11 candidate reference genes. 23 distinct tissues of loquat were tested to show single peak for each primer pair at a specific annealing temperature.

## Abbreviations

18S: 18S Ribosomal RNA
ACT: Actin
DBA: Days before anthesis
DPA: Days past anthesis
GAPDH: Glyceraldehyde-3-phosphate dehydrogenase
HIS3: Histone H3.3
PIP2: Plasma intrinsic protein 2
RG: Reference gene
RPL4: Ribosomal protein L4
RPL18: Ribosomal protein L18
RT-qPCR: Quantitative real-time polymerase chain reaction
SAMDC: S-Adenosyl methionine decarboxylase
TIP41: TIP41-like family protein
TUA3: Alpha-tubulin-3
UGPase: (UDP)-glucose pyrophosphorylase
