# Supplementary material for: Selection of the optimal reference genes for expression analyses in different materials of Eriobotrya japonica
Source: Plant Methods. 2019 Jan 28;15:7. doi: 10.1186/s13007-019-0391-2 (PMC6348664; doi:10.1186/s13007-019-0391-2)
Supplement: Supplementary file 1 — Additional file 1: Table S1. Samples used for reference gene selection. Table S2. Primer sequences for reference gene isolation. Figure S1. Melt curve analysis of the selected 11 candidate reference genes. 23 distinct tissues of loquat were tested to show single peak for each primer pair at a specific annealing temperature. [file 13007_2019_391_MOESM1_ESM.docx]

**Table S1** Samples used for reference gene selection

| **Sample sets** | **tissue** | Developmental phase |
| --- | --- | --- |
| **Fruits** | Fr1, | Receptacle 21 days before anthesis |
|  | Fr2 | Receptacle at anthesis |
|  | Fr3 | Young fruit 56 days past anthesis |
|  | Fr4 | Young fruit 102 days past anthesis |
|  | Fr5 | Mature fruit 126 days past anthesis |
| **Floral tissues** | An | Anther, 1 day before anthesis |
|  | Fi | Filament, 1 day before anthesis |
|  | Fl | Complete flower at anthesis |
|  | If | Inflorescence |
|  | Pe | Petal at anthesis |
|  | Pollen | Pollen |
|  | St | Stigma, 1 day before anthesis |
| **Ovule and seed** | Ov1 | Ovule, 4 days before anthesis |
|  | Ov2 | Ovule, 1 day before anthesis |
|  | Ov3 | Ovule at anthesis |
|  | Seed1 | Ovule during seed set, at 3 days past anthesis |
|  | Seed2 | Young seed at 77 days past anthesis |
|  | Seed3 | Mature seed at ripening |
| **Vegetative tissues** | Ca | Callus |
|  | Ml | Mature leave |
|  | Mst | Mature stem |
|  | Rt | Root |
|  | Yst | young stem |

**Table S2** Primer sequences for reference gene isolation

| **Gene** | **Gene description** | | **Genebank ID** | **Primer sequence (5'-3')** | **Amplicon Length (bp)** |
| --- | --- | --- | --- | --- | --- |
| *RPL4* | Ribosomal protein L4 | | MH196506 | F: AAGGGATACGTGCTGCCG | 381 |
|  |  | |  | R: TCACTGGGCAACCCCG |  |
| *RPL18* | Ribosomal protein L18 | | MH196507 | F: GTGAAGAAAAGCAACGGA | 354 |
|  |  | |  | R: CTTGTATGTCGTCTTGAGC |  |
| *HIS3* | Histone H3.3 | | MH196508 | F: TCGCTACCAAGGCTGC | 339 |
|  |  | |  | R: CACCCCTGATCCTCCTG |  |
| *TUA3* | Alpha-tubulin-3 | | MH196509 | F: TGGGAGCTCTACTGCCTTG | 285 |
|  |  | |  | R: CACAATGTCCTTCCCCACTG |  |
| *SAMDC* | s-Adenosyl methionine decarboxylase | | MH196510 | F: GGTGGGATATGATTTCAAAGATG | 314 |
|  |  |  |  | R: CCAGATCTATCTCTTCAACTTCC |  |
| *TIP41* | TIP41-like family protein | | MH196511 | F: AAGTGAGAGTCATGCCGAG | 279 |
|  |  | |  | R: TACCAGGTACCTTTAGCTTTTG |  |
| *UGPase* | (UDP)-glucose pyrophosphorylase | | MH196512 | F: AAGTGGATGGGGTCAAAGTTC | 334 |
|  | |  |  | R: ACACCAGCACCGAACC |  |


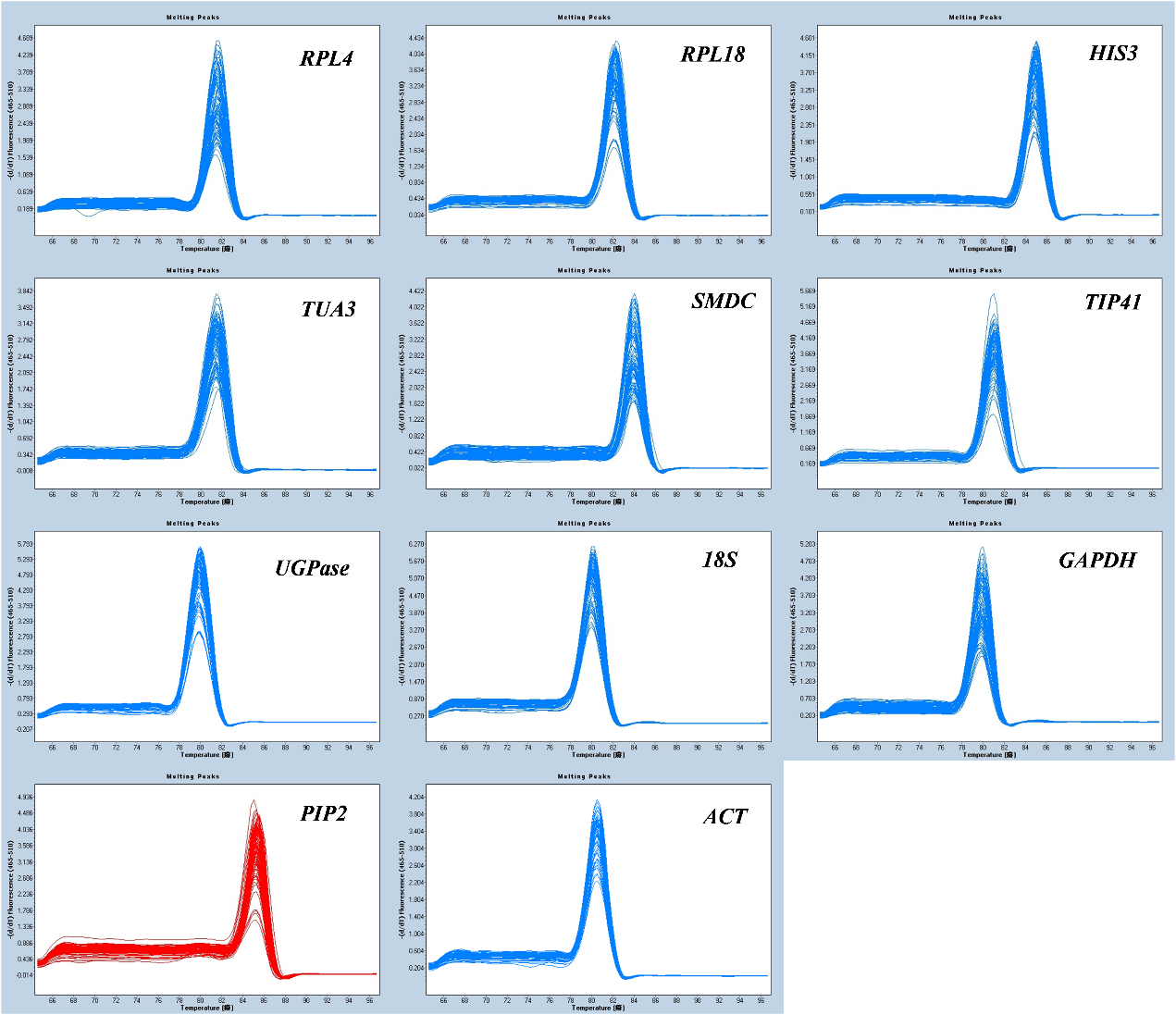


**Figure S1** Melt curve analysis of the selected 11 candidate reference genes.

23 distinct tissues of loquat were tested to show single peak for each primer pair at a specific annealing temperature.
